# Learning effect of computerized cognitive tests in older adults

**DOI:** 10.1590/S1679-45082014AO2954

**Published:** 2014

**Authors:** Rafaela Sanches de Oliveira, Beatriz Maria Trezza, Alexandre Leopold Busse, Wilson Jacob-Filho

**Affiliations:** 1 Universidade de São Paulo, São Paulo, SP, Brazil

**Keywords:** Elderly, Neuropsychological tests, Learning, Diagnosis, computer assisted

## Abstract

**Objective:**

To evaluate the learning effect of computerized cognitive testing in the elderly.

**Methods:**

Cross-sectional study with 20 elderly, 10 women and 10 men, with average age of 77.5 (±4.28) years. The volunteers performed two series of computerized cognitive tests in sequence and their results were compared. The applied tests were: Trail Making A and B, Spatial Recognition, Go/No Go, Memory Span, Pattern Recognition Memory and Reverse Span.

**Results:**

Based on the comparison of the results, learning effects were observed only in the Trail Making A test (p=0.019). Other tests performed presented no significant performance improvements. There was no correlation between learning effect and age (p=0.337) and education (p=0.362), as well as differences between genders (p=0.465).

**Conclusion:**

The computerized cognitive tests repeated immediately afterwards, for elderly, revealed no change in their performance, with the exception of the Trail Making test, demonstrating high clinical applicability, even in short intervals.

## RESUMO

**Objetivo:**

Avaliar o efeito da aprendizagem nos testes cognitivos computadorizados em idosos da comunidade.

**Métodos:**

Estudo transversal, com 20 idosos - 10 mulheres e 10 homens, com média de idade de 77,5 (±4,28) anos. Os voluntários realizaram duas séries em sequência de testes cognitivos computadorizados e seus resultados foram comparados. Os testes aplicados foram: *Trail Making* A e B, *Spatial Recognition*, *Go/No Go*, *Memory Span*, *Pattern Recognition* e *Reverse Memory Span*.

**Resultados:**

Houve efeito de aprendizagem na comparação dos resultados somente no teste *Trail Making A* (p=0,019). Os demais testes não apresentaram alteração significante no desempenho. Não houve correlação entre o efeito de aprendizagem e a idade (p=0,337) e a escolaridade (p=0,362), e nem diferença entre os gêneros (p=0,465).

**Conclusão:**

Os testes cognitivos computadorizados repetidos por idosos imediatamente após sua realização não revelaram alteração do seu desempenho, com exceção do teste *Trail Making*, que demonstrou alta aplicabilidade clínica, mesmo em intervalos curtos.

## INTRODUCTION

With the growth of the elderly population, the incidence of cognitive decline also decreases, causing diseases of great impact on public health. The development of therapeutic techniques and early detection of cognitive decline are very important for maintaining quality of life of the individuals for the longest time possible.

The development of technology has facilitated the adoption of rapid and efficient measures, for both diagnosis and treatment. Computer science enabled the use of this technology in the application of cognitive tests.

Computerized cognitive tests were introduced during the 1970s and gained popularity as the use of computers grew. During the 1980s, several studies were carried out on the advantages and disadvantages of evaluating cognition by these tests. Currently, the studies focus on the development of cognitive batteries capable of evaluating cognitive functions and confirming the efficacy of the existing tests.^(1)^

In order to be used in clinical practice or studies, a cognitive evaluation tool should be validated, that is, it should be capable of assessing the qualities desired, show inter-rater and test-retest reliability, besides maintaining stability when applied by different interviewers and on the same individual at different time points.^(2)^

Among the advantages of using computerized tests, are the capacity to evaluate multiple cognitive functions, greater global consistency and sensitivity, standardization of evaluations, precisely recording the response speed, a more accessible cost, the possibility of issuing an automatic report, and less need for professional training for test application, in which some of the test batteries are self-applicable.^(3,4)^ Nevertheless, in applying this form of evaluation, the behavior of the one evaluated is not analyzed, with his/her reactions or verbalizations, which differs from the neuropsychological assessment traditionally used.^(5)^

Wild et al.^(1)^ point towards an inherent limitation of computerized cognitive tests, especially when used with the elderly population, which is the lack of psychometric data, such as data reliability and validity, in comparison with the traditional measurements, paper and pencil. Several researchers had the objective of verifying the equivalence between traditional and computerized tests. Studies by Collerton et al.^(6)^ and Wagner and Trentini^(7)^ observed the equivalence between the two methods; whereas the studies by Feldstein et al.^(8)^ and Steinmetz et al.^(9)^ showed a difference in the results suggesting that skills to use computers might favor a better performance of the person assessed. The study done by McDonald et al.^(10)^ showed a yet greater difference in the elderly population.

The repetition of cognitive tests, which is very common in neuropsychological clinical practice, also shows a variation in the results found, suggesting an effect of learning when compared to the results of serial applications.^(11,12)^

The effect of learning or of practice is defined as improvement in performance of the test by volunteer, without having been offered any intervention or condition that could justify it. Various reasons have been discussed to explain the gains in scores induced by practice, such as reduced anxiety or increased familiarity with the testing environment and procedural learning.^(13)^ The studies that do not consider the effect of learning on the repetition of tests may lead to wrong conclusions on the benefits of interventions and even mask the presence of cognitive decline, primarily in the elderly population.^(14)^

In a meta-analysis on the effects of learning in neuropsychological tests, Calamia et al.^(15)^ found few studies in the literature comparing the performance of elderly individuals.

## OBJECTIVE

To confirm the applicability of computerized tests in elderly individuals of the community, to verify the possibility of repeating them immediately with no modification in performance, and to evaluate the effect of learning these tests in the elderly of the community.

## METHODS

The sample of 20 aged individuals was selected by convenience between June and October 2012, from the Outpatient's Clinic - Department of Geriatrics, *Hospital das Clínicas da Faculdade de Medicina da Universidade de São Paulo* (HCFMUSP).

The inclusion criteria were score on the Mini Mental Status Examination^(16)^ within normality for schooling level;^(17)^ score of the Geriatric Depression Scale −15 ≤5 points;^(18)^ being literate; presenting with sensory functions (visual and auditory) that enable the performance of the tests; and agreeing to participate in the study, by signing the Informed Consent Form.

The exclusion criteria were the use of five or more medications; habitual ingestion of alcoholic beverage; and presenting with decompensated or symptomatic systemic disease.

The first 20 individuals selected responded to a socioeconomic and clinical evaluation protocol, and then were submitted twice to a battery of computerized cognitive tests.

The first evaluation (T1) had the objective of presenting the tests, which were reapplied (T2) in the same sequence after the end of the first battery.

The time used for each battery was approximately 30 minutes.

This study was approved by the Ethics Committee for Analysis of Research Projects (CAPEPesq) of the HCFMUSP, under number 149,925, on November 21st, 2012, and was a part of the thematic project Human biometeorology: analysis of the effects of environmental variables (meteorological, thermal comfort, and air pollution) and of climate changes in the geriatric population of the city of São Paulo, financed by The State of São Paulo Research Foundation (FAPESP), process number 2010/10189-5.

### Cognitive evaluation

A series of computerized tests was applied that was developed at the Universities of Stanford, San Francisco, and McGill.^(19)^ The series was composed of simple and quick tests performed by touch screen.

The tests selected were:

– Trail Making A (TMA): it evaluates attention relative to time to perform the task. It is composed of circles numbered from 1 to 25, randomly distributed, which should be touched in the growing order of the numbers. When circles are touched in the correct order the color changes indicating that the patient may proceed with the task. If the option touched is wrong, an “x” appears over the number, allowing the choice of another option;
– Trail Making B (TMB): similar to the previous test. It is composed of circles containing 13 numbers and 12 letters, randomly distributed, which should be touched in the increasing order of the numbers and letters of the alphabet (1A, 2B, 3C);
– Spatial Recognition: it evaluates spatial recognition relative to the number of correct answers. It consists of visualizing, for each time frame, five squares of the same color and size in certain positions on the screen. The patient should memorize the position of the squares. Next, two squares appear at the same time, one of them in a new position and the other in the original position, which should be touched by the volunteer;
– Go/No-Go: it evaluates the reaction time and consists of the presentation of the figure of a fruit. The volunteer should click on the space key as quickly as possible every time he/she visualizes the figure of this fruit, as figures of other fruits are randomly presented. The time for reaction and number of errors are evaluated;
– Pattern Recognition: it evaluates memorization of details and orientation of figures by means of the number of correct answers. The test comprises a sequential presentation of 12 images, which should be memorized by the volunteers in the details and positions on the screen. Next, by pairs, a new figure and an original figure are presented, which should be touched by the volunteer;
– Memory Span: evaluated spatial memory by means of presentation of ten cards of the same color on the screen. The cards change color individually, following a determined sequence. The volunteer should next repeat the order of change of the cards, touching them on the screen. If two sequences are identified correctly, one unit is added to the number of cards that change color. The test evaluates the number of correct sequences;
– Reverse Memory Span: similar to the previous test, but the cards should be touched in inverse order of their color change.

The results were presented by means, standard deviations, and proportions. The Friedman and Wilcox tests were used, considering that the data were paired, in order to compare the two collection times for all the variables analyzed. To relate the data with the sociodemographic data, Spearman's correlation and Student's *t* test were used, considering the significance level of p<0.05.

## RESULTS

The clinical and sociodemographic profile of the 20 elderly individuals (10 women and 10 men) is detailed on table 1. We highlight that in women, the mean age was 69.7 (±4.85) years, and for men, 74.7 (±3.46) years, with p=0.172, while the schooling level for women was 8.5 (±4.27) years and for men, 7.0 (±4.37), with p=0.160.

**Table 1:** Sociodemographic characteristics of the sample studied (n=20)

Descriptors		Mean (SD)	n (%)
Schooling (years)		7.75 (4.28)	
Age (years)		72.20 (4.84)	
BMI		25.27 (3.54)	
MMSE (points)		28.35 (1.23)	
GDS (points)		1.20 (1.28)	
Gender			
	Female		10 (50)
	Male		10 (50)
Marital status			
	Married		10 (50)
	Widowed		4 (20)
	Single		2 (10)
	Divorced		4 (20)
Religion			
	Believes in God		20 (100)
Social security status			
	Worker		3 (15)
	Retired		17 (85)
Smoker			
	No		18 (90)
Engagement in physical activity			
	Yes		11 (55)
Use of a computer			4 (20)
Use of automated teller machine			5 (25)

SD: standard deviation; BMI: body mass index; MMSE: Mini Mental State Examination; GDS: Geriatric Depression Scale.

In comparing the cognitive function tests performed in sequence, a significant difference was noted only in the TMA test (Table 2).This result observed in the TMA Test did not correlate with age (p=0.337) and schooling (p=0.362) according to Spearman's test, even when comparing the percentages of learning (T2-T1/T1). Student's *t* test also showed no significant difference between the percentage of learning of men and women (p=0.465).

**Table 2:** Comparing results of cognitive function tests T1 and T2

Tests	Mean (SD) n=20		p value*
	T1	T2	
Trail Making A (s)	85.4 (33.6)	72.8 (21.0)	0.019
Trail Making B (s)	168.0 (68.6)	160.6 (63.9)	0.550
Spatial Recognition	2.95 (1.23)	3.15 (0.93)	0.450
Go/No Go (s)	583.2 (115.0)	555.9 (84.8)	0.601
Go/No Go	0.3 (0.571)	0.4 (0.598)	0.317
Memory Span	3.70 (0.80)	410 (1.15)	0.163
Reverse Memory Span	3.15 (0.99)	3.15 (1.23)	0.869
Pattern Recognition	8.45 (1.99)	8.30 (1.92)	0.700

* Friedman test.

SD: standard deviation; (s): seconds.

## DISCUSSION

Our data demonstrate a significant improvement in the TMA results between the two evaluations. Recent studies have shown that the improvement in performance, brought about by the effect of practice may remain from one to six weeks^(20)^ and is no longer significant one to seven years after the initial evaluation.^(21)^

Few studies, however, have evaluated the effect of learning on computerized cognitive tests. Raymond et al.^(22)^ applied a battery of computerized tests (MicroCog) and found significant effects of learning when applying it twice, with an interval of two weeks. The improvement in performance was attributed to increased confidence in use of a computer.

A study carried out by Beglinger et al.^(23)^ with healthy adults who used drugs to improve cognitive function showed better computerized test scores even in individuals who did not undergo drug intervention throughout the six repetitions, showing a high level of learning in TMA, especially in the third and fourth repetitions. The same gain in performance was not observed in TMB results, or in the present study.

It is believed that, in this study, the fact of the tests being computerized might have increased the degree of anxiety and the fear of inability to conclude the test, regardless of the socioeconomic characteristics. The fact that the population evaluated comprised exclusively elderly individuals also contributed towards increased anxiety, since this age group historically utilizes computers less frequently.^(24)^ In this study, the use of computers was only reported by a small portion (20%) of the sample. It is important to point out that the TMA was the first test to be performed, a fact that could have compromised the results of the first evaluation, considering a significant difference in the comparison between the two performances. Lezak et al.^(25)^ stated that after the first test, the strategies developed to master the task could facilitate the performance of the other evaluations as of the first application, reducing the percentage of progression when comparing the results of both assessments. This fact contributed towards the absence of the effect of significant learning in the other tests applied, showing that after handling of the computer was introduced, the volunteers felt greater ease in performing the tests.

Some authors reported that in comparing the effect of learning between the young and old, ageing makes performance in evaluations similar, but the factors that lead the elderly population to show a smaller effect are still not clear.^(26)^ The smaller capacity for memorization and coding of relevant information in the initial tests may be a cause for such a finding.

Irrespective of the time interval between evaluations, and even of the volunteers´ age, some individual factors, such as motivation at the initial evaluation, satisfactory clinical conditions, a high intelligence coefficient, and high schooling level, may favor the effect of learning.^(27,28)^

A study carried out with TMA and TMB in their traditional versions, showed no difference among individuals of different age groups.^(29)^ It is believed that the tests used here, whether due to low complexity or the furnishing of appropriate instructions, made the performance of elderly individuals easier, regardless of some factors, such as age and schooling level.

Duff et al.^(30)^ found no association between the sociodemographic data (gender, schooling, and age) and intensity of the effect of learning on traditional cognitive tests in 268 adults, which is in agreement with the results of this study.

A large part of the studies aiming to verify the effect of learning in the elderly was carried out in adults, considering the mean age was approximately 50 years. Even when designed to evaluate the impact of age on this phenomenon.

Although most studies are performed with tests in their traditional form (paper and pencil), it is believed that the introduction of computerized tests at this age is important, and that these individuals should increasingly use this piece of equipment.

Rarely is the immediate effect of learning evaluated; nevertheless, we assume here that this model of evaluation allowed us to detect the inexistence of this phenomenon in this set of cases. We believe that the immediate repetition of most of the tests used was not capable of producing a gain in performance, but this should not happen later, a fact that can be corroborated by new studies or by an increase in the number of cases.

## CONCLUSION

Computerized cognitive tests are performed by elderly individuals regardless of their prior experience with this technology. Such tests can be repeated immediately and with no interference in performance.

In the Trail Making A test, which is the one with least complexity among all tests applied, demonstrated that there was learning. In the sample studied, there was no difference in the other computerized tests applied (Trail Making B, Spatial Recognition, Go/No-Go, Pattern Recognition, Memory Span, and Reverse Memory Span), demonstrating high clinical applicability, even with short intervals.
